# The First Confirmed Outbreak of Recombinant Myxomatosis Virus in the Brown Hare (*Lepus europaeus* P.) Population in Hungary

**DOI:** 10.1155/tbed/3360897

**Published:** 2026-07-09

**Authors:** A. Bende, A. Hegyi-Nándori, R. László, M. Marsai, A. Örkényi, P. Malik, F. Jánoska, L. Bánáti

**Affiliations:** ^1^ Institute of Wildlife Biology and Management, University of Sopron, Sopron, Hungary; ^2^ National Food Chain Safety Office, Veterinary Diagnostic Laboratory Directorate, Virology Department, Budapest, Hungary; ^3^ National Food Chain Safety Office, Veterinary Diagnostic Laboratory Directorate, Pathology Department, Budapest, Hungary

**Keywords:** European brown hare, ha-MYXV, Hungary, *Lepus europeaus*, myxoma virus, species jump

## Abstract

We investigated the emergence and spread of the ha‐MYXV (recombinant myxoma virus) in the brown hare (*Lepus europaeus*) population in Hungary. During PCR genetic analysis of 37 samples collected between October 7 and 28, 2025, at the beginning of the outbreak, we found recombinant myxoma virus DNA in 89.2% of the samples, which are the first known ha‐MYXV type epidemic infections in brown hares in Hungary. The recombinant MYXV infecting Lagomorpha species first entered western Hungary as a result of an outbreak near Vienna (Austria), and then appeared in the eastern part of the country within a week. Based on European experience, the new virus strain will have a significant impact on the Hungarian populations of brown hares, which calls into question the possibility of profitable game management with this species in many areas, foreshadowing the need for nationwide monitoring system studies to map the epidemiological characteristics of recombinant MYXV.

## 1. Introduction

The population of brown hares has declined dramatically in Hungary in recent decades. Compared to the 1960s, the population has now been reduced to about one‐third (*n* = 536,500 individuals), yet this species still plays a significant role in game management [1–4]. In the 2024–2025 hunting year, the total hunting utilization of this species (including live capture and shooting) amounted to 157,441 individuals, of which 127,938 brown hares were harvested (shot) and 29,500 individuals were captured alive [2]. The captured individuals were typically exported abroad (to Italy, France, and Croatia), while no live hares were imported into Hungary [5].

The main factor determining the decline in population is the drastic transformation of the agricultural environment, with the development of intensive, large‐scale agriculture from the late 1940s onwards, which has significantly reduced the optimal habitat of the brown hare [6]. According to the more negative scenarios, if current trends continue, the future population of brown hares will soon fall below the critical population density of four individuals per 100 hectares in a significant part of the country, at which point sustainable hunting is no longer possible [1]. In addition to significant habitat transformation and habitat loss, the decline in population is also due to the lack of predator control measures [7], changes in weather conditions [8], increased use of insecticides [9, 10], an increase in deaths due to collisions with motor vehicles [11], and the emergence of numerous infectious diseases [12–14], among which the emergence of the new ha‐MYXV disease, causing drastic mortality events, poses a serious threat to the Hungarian brown hare populations. In certain areas, overexploitation may contribute to declines in Hungarian populations of the species; however, hunting has not been the primary factor responsible for the decrease in brown hare populations. The active investigation of changes affecting the brown hare population and the monitoring of the impact of ha‐MYXV are of particular importance as the brown hare plays a significant role in Hungarian small game management. Hungary has a stable market background both in terms of live hare export and hunting [1]; therefore, this issue is of considerable economic relevance. Particular emphasis should be placed on elucidating the rate of spread and the factors influencing transmission (including the role of vectors and human‐related risk factors) in order to formulate evidence‐based recommendations for hunting right holders aimed at mitigating disease‐associated losses. At the same time, it is also crucial from an ecological perspective since the brown hare constitutes an important component of the food web. It is a primary prey species for several protected raptor species, such as the Eastern imperial eagle [15].

MYXV was first reported in Uruguay in 1896, where it caused significant losses only in *Oryctolagus* species, while subclinical symptoms and well‐defined skin fibromas were reported in the common tapeti (*Sylvilagus brasiliensis*) [16–18]. The virus was first introduced to Europe in France in 1952 with the aim of controlling the significantly overpopulated European rabbit (*Oryctolagus cuniculus*) population [17], but the pathogen then became endemic throughout Europe [19, 20]. It was first detected in Hungary in 1959 [21]. Although Bull and Dickinson [22] did not find any sick individuals during artificial vaccination experiments conducted in Australia in the 1930s, serological results confirmed its presence in France [23–25], Ireland [26, 27], Great Britain [28], and Italy [29], indicating that MYXV can occasionally infect *Lepus* species. This virus, which causes endemic disease spread by ectoparasites—especially mosquitoes—has been present in Europe for more than 50 years [30], but no epidemic outbreaks have been recorded in brown hares to date [28, 31, 32]. The first epidemic outbreak of MYXV inan Iberian hare (*Lepus granatensis*) population was reported in Spain, on the Iberian Peninsula, in 2018 [33], followed by outbreaks in Portugal [34], in 2023 in Germany, and in 2024 in several provinces of the Netherlands [35, 36]. This transfer between species belonging to the *Orctolagus* and *Lepus* genera was the result of the emergence of a recombinant myxoma virus strain, ha‐MYXV [34]. The new virus reached Bulgaria in 2024 [37], then Austria in 2025 [38], the Czech Republic and Slovakia [39–41], and, from Austria, Hungary (Figure 1), posing a new challenge to the Hungarian wildlife management sector and animal health organizations [42].

**Figure 1:**
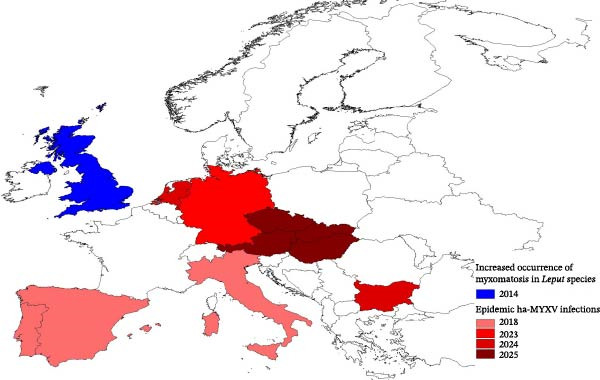
The temporal occurrence of the ha‐MYXV strain in Europe, which also infects brown hares.

## 2. Methodology

### 2.1. Study Area and Sample Collection

Since approximately 127,900 brown hares were bagged in Hungary in 2024 [2], sampling opportunities were not limited, but we necessarily concentrated on areas where an outbreak was suspected. Sampling was carried out between October 7 and October 28, 2025, in Hungary, in habitats of particular importance for brown hares in the LittleHungarian Plain (Győr‐Moson‐Sopron county) and Great Hungarian Plain (Jász‐Nagykun‐Szolnok, Békés, Csongrád‐Csanád, Hajdú‐Bihar counties), where significant hare mortality had been observed. The samples were collected partly through passive surveillance one specimen, that is, by collecting carcasses, and partly during the hunting season (October 1 to December 31, Decree 79/2004 (V. 4.) of the Ministry of Agriculture), with 15 specimens obtained through hunting [43]. In this case, sampling was conducted in a targeted manner in those counties and hunting areas where, based on reports from game managers, there was a strong suspicion of ha‐MYXV infection. For the part of the samples examined (*n* = 21) that were submitted by those authorized to hunt, they were likewise collected during hunting activities at the beginning of the hunting season; however, these cases were data‐deficient, as neither age nor body mass was recorded. For laboratory examinations, the carcasses of both hunted and found individuals were submitted in their entirety for analysis. The sampling location was specified using the municipal boundaries at the time of the sampling. This means that, in accordance with the National Food Chain Safety Office sample submission protocol, the sampling location was recorded as the nearest settlement to the site where the carcass of the deceased individual was found or where the animal was harvested during hunting. In addition to localization, we also recorded the age and macroscopically visible lesions of the individuals we collected (*n* = 16). Age determination was performed as described by Pepin [44], that is, by feeling the epiphyseal cartilage bulge on the surface of the radius extremitas distalis. The cartilage bulge is clearly detectable up to the age of 8–9 months but cannot be felt thereafter [45]. The former individuals are younger than 9 months (I. juv.), while the latter are older than 9 months (II. ad.) [44, 45].

### 2.2. Laboratory Tests

Brown hares (*n* = 37) from the respective hunting grounds were examined in the laboratories of the National Food Chain Safety Office, Veterinary Diagnostic Laboratory Directorate, Pathology, and Virology Department. Laboratory analyses were conducted in Budapest, Hungary.

#### 2.2.1. Pathology and Histopathology

Necropsies were performed on all submitted cases. Following the postmortem examination, tissue samples from the spleen, liver, kidneys, lungs, heart, brain, and affected skin areas were collected for histopathological examination. The tissue samples were fixed in 10% buffered formalin, embedded in paraffin, and then stained with hematoxylin and eosin (H&E) in 5 μm thick sections.

#### 2.2.2. Nucleic Acid Extraction

Viral DNA was isolated from 25–30 mg of tissue, all of which were fresh, non‐preserved samples collected from all carcasses (*n* = 37). While during the pathological examination different organs were sampled from each animal, the nucleic acid extraction was made from the mix of the organs of each individual hare. As a first step, tissue samples were placed in sterile 2 mL Eppendorf tubes with stainless steel balls and supplemented with 1 mL of phosphate‐buffered saline (PBS) (pH 7.4). Homogenization was performed using a TissueLyser II device (QIAGEN) at a frequency of 25 Hz for 2 min. After homogenization, the samples were centrifuged at 7000 × *g* for 5 min. Nucleic acid extraction was performed from 200 µL of supernatant using a commercially available DNA extraction kit (MagnifiQ 96 Pathogen instant kit, A&A Biotechnologies) according to the manufacturer’s protocol using a Kingfisher Flex (Thermo Fisher Scientific) nucleic acid isolation device. The extracted DNA samples were stored at −20°C until PCR testing.

#### 2.2.3. Myxomatosis‐Specific Multiplex Real‐Time PCR Examination

For rapid detection of infectious myxoma virus and differentiation between classic MYXV and ha‐MYXV strains, we used a real‐time multiplex PCR system developed by Abade dos Santos et al. [46]. The system is capable of simultaneously amplifying the m000.5L/R duplicated gene, the m009L gene, and certain sections of the m060L gene, thereby enabling differentiation between the two strains.

The basis for differentiating the myxoma virus strains is an insertion within the genetic material of the recombinant strain (ha‐MYXV) compared to that of the classical myxoma virus. During this process, a 2.8 kilobase‐pair (kbp) sequence was integrated into the m009L gene segment.

Among the three qPCR systems, the m000.5L/R system (FAM channel) is specific to the m000.5L/R gene located in the inverted terminal repeats (ITRs) at both ends of the viral genome. Since this gene is highly conserved in all myxoma virus strains (whether classical or recombinant), it serves as the method for general viral detection.

The system specific to the m009L (Cy5 channel) gene segment was used to identify classical MYXV strains. In recombinant strains (ha‐MYXV), a 2.8 kbp insert is located at this point, which disrupts the gene; consequently, the distance between the primers becomes too great, and the probe cannot bind, preventing amplification.

Finally, the m060L system (HEX channel) specifically targets a segment of the m060L gene present in recombinant strains (ha‐MYXV). This gene is part of the inserted 2.8 kbp sequence; therefore, it yields a positive signal only in the case of the presence of the recombinant virus.

The sequences of the primers and probes used are summerized in Table 1, and the expected results in the case of infection with the different MYXV strains can be found in Table 2.

**Table 1:** Primers and probes used in the myxomatosis‐specific multiplex real‐time PCR test.

Oligomer	Nucleotide sequence (5^′^‐3^′^)		
Forward primer	CGACGTAGATTTATCGTATACC	TCCATTTACGATACACGCCGACGC	GATTCTTTAATCTGGTTGAGGCAACTA
Reverse primer	GTCTGTCTATGTATTCTATCTCC	ACAACGTTCTATACTGTTTAGGGGGTACG	GGATATTATTACGCTCCATTATCGGAGG
Probe	TCGGTCTATCCTCGGGCAGACATAGA	TACGATCTACTGACGAACGAATACAGTTTAATGCC	CTGATAAGTACCCCTTATCTACAAAAACGGGTG

**Table 2:** The explanation of possible results in the presence of classical/recombinant MYXV strains.

Type of infection	Amplification and fluorescence detection		
	*m000.5L/R* FAM	*m009L* Cy5	*m060L* HEX
MYXV	Yes	Yes/no^a^	No
ha‐MYXV	Yes	No	Yes
MYXV and ha‐MYXV (coinfection)	Yes	Yes/no^a^	No
Non‐infected	No	No	No

^a^The m009L system may lack the detection of some classical MYXV strains (e.g., some vaccine strains)

For the reactions, we used Clara Probe Mix No‐ROX (PCRBIOSYSTEM, PB20.63) enzyme according to the manufacturer’s recommendations. During PCR, we used 4 µL of template in a final reaction volume of 20 µL. Each run included a confirmed positive and negative control for the myxoma virus, but we haven’t used any controls for the validation of the nucleic acid extraction. The system to monitor nucleic acid extraction efficiency is already available and will be implemented in future studies.

The final concentrations of primers and probes are summarized in Table 3, while Table 4 contains the amplification protocol of the multiplex qPCR method.

**Table 3:** The final concentrations of primers and probes used.

Concentrations (nM)			
Gene	Forward primer	Reverse primer	Probe
*m000.5L/R*	500	500	250
*m009L*	500	500	250
*m060L*	500	500	250

**Table 4:** The amplification protocol of the multiplex real‐time PCR.

PCR phases	Amplification protocol		
	Temperature (°C)	Time	Cycle
Polymerase activation	95	3 min	1
Denaturation	95	15 s	40
Anneal/extension	60	30 s	

## 3. Results

During pathological and histological examinations, we observed typical nodular edematous lesions characteristic of myxomatosis infection nodular lesions with edema and dermatitis on the head, around the eyes (swelling and conjunctivitis) and nose, as well as on the legs and in the anogenital region, which corresponds to the symptoms characteristic of poxvirus infection (Figure 2). Conjunctivitis is accompanied by severe tearing and myxedema swelling around the eyes, which can lead to blindness in the final stages. The above‐described clinical signs—namely, focal, edematous lesions, and conjunctivitis—were observed in all individuals confirmed to be infected with the virus; only minor to moderate individual variation was noted in the severity of the lesions. In addition, congestion of the lungs, mild pulmonary hemorrhage, and alveolar edema were also observed. All of these are typical symptoms of classic myxomatosis. Loss of body condition is an important indicator in the context of ha‐MYXV infection, and although we were only able to examine a relatively small sample size (*n* = 11), for these individuals, we recorded not only age but also body mass data (Table 5).

**Figure 2:**
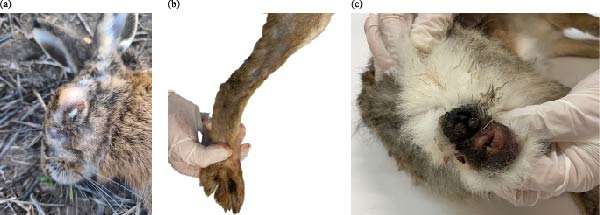
Skin lesions of a brown hare affected by myxomatosis: edematous eyelids, lips and nose, conjunctivitis (a), myxomatous skin nodules on the legs (b), and typical anogenital edema (c) (Fotó: G. Keszthelyi and A. Bende).

**Table 5:** Age and body mass data of ha‐MYXV confirmed infected hares.

Number of samples	Sampling location	Age	Body mass (g)
1	Abertkázmérpuszta	ad	3375
2		ad	4865
3		ad	3680
4		ad	4020
5		ad	3310
6		ad	3115
7	Bogyoszló	ad	3295
8		ad	3225
9		juv	1410
10		ad	3350
11		ad	2990

The mean body mass of the adult subsample was 3522.5 g (SD = 553.89). However, the age data reflect a marked imbalance as 90.9% of individuals of known age were adults. The assessment of body condition and the age distribution of infected individuals should therefore be addressed in future studies based on larger sample sizes.

The microscopic skin lesions were eosinophilic intrascytoplasmic inclusions in the keratinocytes in the skin stroma and epidermal hyperplasia characteristic of myxomatosis in the epithelial tissue. Lymphocyte depletion could also be found in the spleen and in the lymph nodes (Figure 3).

**Figure 3:**
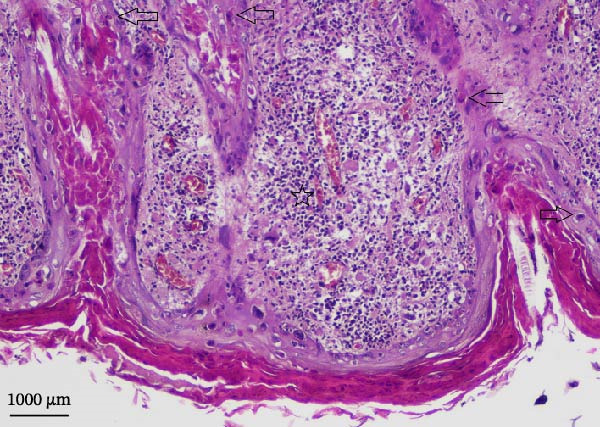
Epidermal hyperkeratosis, inflammation (asterisk) in the dermis, and intracytoplasmic eosinophilic inclusion bodies in the keratocytes (arrows). H&E stain. x100.

A total of 37 collected samples were tested using a multiplex PCR system. We considered those samples positive whose specific sigmoid amplification curve exceeded the set threshold on the given channel before the 40th cycle, while we interpreted all samples that did not have a Cq value (quantification cycle) as negative. During the tests, with the exception of the samples from Püspökladány (2) and Magyarcsanád (1), we observed a specific amplification curve in the FAM (m00.5L/R gene) and HEX (m060L gene) channels, while all samples gave negative results on the Cy5 (m009L gene) channel. The results are consistent, and as the fluorescent sign was present in both the FAM and HEX channels, it indicates the presence of the recombinant strain in these samples.

In all PCR runs, the Cq value of the positive control did not exceed 25, and the negative control did not exceed the set limit in any case, so all runs can be considered valid (Table 6). Based on the above, out of the 37 individuals examined, the presence of ha‐MYXV was confirmed in 33 cases, representing 89.2% of the analyzed sample.

**Table 6:** Origin, date of submission, PCR test results, and clinical symptoms of the samples examined.

Date	County	Origin	Number of sampled animals	Results of multiplex qPCR	Ct values (FAM channel)	Ct values (HEX channel)	Ct values (CY5 channel)	Variant type according to PCR	Clinical symptoms
07.10.	Győr‐Moson‐Sopron	Hegyeshalom	1	All samples positive	25.13	24.93	>40	Recom‐binant	Purulent conjunctivitis
13.10.	Jász‐Nagykun‐Szolnok	Karcag	4	All samples positive	**1**: 30.56, **2**: 31.79, **3**: 29.75, **4**: 31.15	**1**: 32.64, **2**: 33.38, **3**: 31.55, **4**: 33.28	>40	Recom‐binant	Only the sings of the trauma
13.10.	Békés	Dévaványa	1	All samples positive	33.27	34.94	>40	Recom‐binant	—
14.10.	Jász‐Nagykun‐Szolnok	Kisújszállás	1	All samples positive	19.36	18.73	>40	Recom‐binant	—
15.10.	Győr‐Moson‐Sopron	Rábapordány	11	Only eight animals were tested, all animals positive	**1**: 24.31, **2**: 25.52, **3**: 19.11, **4**: 18.07, **5**: 24.65, **6**: 24.38, **7**: 26.28, **8**: 16.95	**1**: 23.55, **2**: 25.46, **3**: 18.61, **4**: 16.88, **5**: 24.42, **6**: 24.32, **7**: 25.89, **8**: 16.25	>40	Recom‐binant	Characteristic skin lesions on the head of three animals, and on the legs of two animals
15.10.	Győr‐Moson‐Sopron	Bogyoszló	5	All samples positive	**1**: 24.74, **2**: 18.26, **3**: 19.24, **4**: 19.77, **5**: 15.69	**1**: 24.14, **2**: 16.20, **3**: 18.28, **4**: 19.06, **5**: 14.78	>40	Recom‐binant	Diffuse skin lesions over the entire body of three animals, and on the heads of two animals
15.10.	Hajdú‐Bihar	Püspökladány	2	All samples negative	>40	>40	>40	—	—
15.10.	Csongrád‐Csanád	Csanytelek	2	All samples positive	**1**: 18.48, **2**: 19.61	**1**: 18.23, **2**: 19.16	>40	Recom‐binant	—
16.10.	Győr‐Moson‐Sopron	Mosonmagyaróvár	3	All samples positive	28.93	28.33	>40	Recom‐binant	Skin lesions
18.10.	Békés	Dévaványa	2	All samples positive			>40	Recom‐binant	Characteristic skin lesions on the head, and on the legs of two animals
20.10.	Csongrád‐Csanád	Magyarcsanád	1	All samples negative	>40	>40	>40	—	—
21.10.	Csongrád‐Csanád	Magyarcsanád	1	All samples negative	>40	>40	>40	—	—
28.10.	Győr‐Moson‐Sopron	Kapuvár	1	All samples positive			>40	Recom‐binant	Swelling/edema around the eyelids and genitalia and hyperkeratosis
28.10.	Hajdú‐Bihar	Hortobágy	2	All samples positive			>40	Recom‐binant	

*Note*: The bold values indicate the serial number of the samples being tested, so that it can be more easily distinguished from the results.

Barely a week passed between the first positive sample registered in western Hungary (October 7, 2025) and the confirmed ha‐MYXV infection in the eastern and south‐eastern regions of the country (October 13, 2025) (Figure 4). Considering the rate and spatial pattern of spread, we can expect the virus to appear in any part of the country.

**Figure 4:**
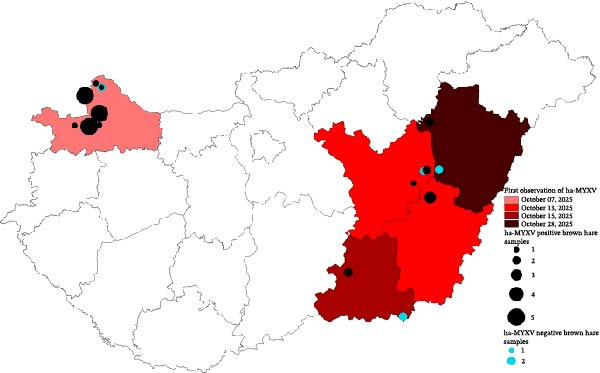
Confirmed cases of recombinant ha‐MYXV in Hungary.

## 4. Discussion

The macroscopic and histological changes we observed in brown hares correspond to those reported in the international literature in connection with ha‐MYXV [18, 28, 30, 32, 34, 37, 39, 46]. Infection with ha‐MYXV typically results in acute or hyperacute myxomatosis, which is also confirmed by the significant loss of condition we observed.

The pattern of the disease’s spread in Europe is radial and points northeast [35]. Based on the spatial and temporal patterns of known infections in Hungary, we assume that the virus reached the country from Austria, but the short‐term appearance of the disease in western and eastern Hungary cannot be explained by arthropod vectors alone. Our results support Fischer’s [35] finding that anthropogenic pathogen transmission is an important factor in the spread of the virus, alongside direct contact (i.e., animal‐to‐animal transmission) and insects (including mosquitoes). Several studies point to the major role of arthropod vectors, which seems to be confirmed by late summer outbreaks [35, 46]. Based on 84 brown hares examined in Bulgaria [37], it was concluded that the spread of ha‐MYXV was not widespread in the populations examined in different parts of the country. In addition, increased contact during the breeding season may represent an important factor; thus, the proportion of infections may rise during the late winter and early spring vector‐free periods. However, we did not find relevant data in the literature addressing this issue.

The extent to which the emergence of the new virus strain will affect brown hare populations remains an open question. For the time being, the morbidity and mortality rates in Hungary are unknown, as are the survival rates. Serological tests may provide some answers to the above questions, although these tests cannot distinguish between exposure to classic MYXV and ha‐MYXV [31]. Although during this study we haven’t performed any serological testing, in an ongoing study, we try to detect antibodies from blood samples taken from brown hares.

The most favorable scenario for the new ha‐MYXV is increased genetic resistance to the disease [47], as was the case with *Oryctolagus* species [16, 48]. However, in our opinion, this process may be limited by the overexploitation of infected populations and populations that have undergone infection and have significantly reduced numbers.

We observed a loss of body condition in the hares from western Hungary (*n* = 10), as the average body weight of infected individuals in the sample restricted to the adult age group was lower than the average of the adult subsample from the same area in previous years (4265 g SD = 360.2 g [1],) by 743.7 g. Further investigation of this issue with a larger sample size would be warranted, as the loss of body condition reported in the literature [28] may represent an important indicator of factors predisposing individuals to disease and increasing the risk of mortality, as well as a relevant parameter in understanding the course and progression of the disease. The imbalance observed in the age distribution has also been reported in the international literature [35], likewise favoring adult individuals. However, this issue can only be examined in a manner that adequately meets the requirements of representativeness on the basis of larger sample sizes.

## 5. Conclusion

The brown hare holds a prominent position in Hungarian game management; therefore, the emergence of ha‐MYXV is of strategic importance for the small game sector. This necessitates the formulation of research‐based guidelines at the early stages of the outbreak. In order to gain a better understanding of this new disease, we consider it necessary to develop a nationwide, wide‐ranging monitoring system that is consistent in terms of time and space, which will help to better understand the dynamics, circulation, and epidemiological characteristics of the new ha‐MYXV in the brown hare population, as epidemiological measures can only be effective if these characteristics are known.

Based on experience, it is plausible that new, more virulent virus recombinations affecting Lagomorph species will emerge, which could have serious consequences for European brown hares as well [31, 46]. Monitoring will play a key role in developing an effective action plan and mitigating the impact of epidemics, which should be implemented with the help of subsidized serological tests, thus motivating those eligible to hunt to join the sampling network and actively collect samples. This is particularly important if there is a sudden increase in the number of brown hare deaths in their area.

Some recommendations regarding the measures can already be formulated prior to the development of the monitoring system and the implementation of official measures, which will help eligible hunters mitigate losses. The spread of ha‐MYXV in brown hare populations cannot be prevented, and although it is theoretically possible to eradicate mosquitoes in areas affected by the epidemic, this is unlikely to have any practical effect on mitigating losses. The vaccination of domestic rabbit populations is particularly important in areas affected by the epidemic. Human transmission also plays a role in the spread of the disease, so it is recommended that live trapping be stopped in order to reduce its spread. Moderate exploitation of the reduced core population that has survived the disease—or, in the case of significant losses, no exploitation at all—can help the hare population in the affected areas recover. It is advisable to dispose of individuals with visible symptoms that have been killed during hunting if this can be done locally. It is not recommended to sell the meat of these animals, although the consumption of animals without symptoms does not necessarily have to be prohibited, but transporting them further away is a concern in terms of the spread of the epidemic. It is important that the carcasses of dead hares found in hunting areas are disposed of by burying them at a sufficient depth.

## Author Contributions

**A. Bende**: concept development, sample collection organization, writing – review and editing, sources. **A. Hegyi-Nándori**: writing – review and editing, laboratory work. **R. László**: review and editing, sample collection organization, research. **M. Marsai**: pathology, histopathology, writing – review and editing. **A. Örkényi**: method optimization, laboratory work, writing, review. **P. Malik**: review and editing. **F. Jánoska**: review and financing. **L. Bánáti**: organization of sample collection, pathology, writing – review and editing.

## Funding

The research underlying this study was carried out with the support of the Ministry of Agriculture in cooperation with the Institute of Wildlife Biology and Management at the University of Sopron, the National Food Chain Safety Office, Veterinary Diagnostic Laboratory Directorate, and the Virology and Pathology Department. This article was supported by the Faculty of Forestry of the University of Sopron.

## Ethics Statement

No ethical approval was required for this study as no tissues were collected from live animals and no live animals were captured or culled for the purpose of sample collection. The sampling of carcasses used in this research does not fall within the scope of the Hungarian Government Decree 40/2013 (II. 14.) on the protection of animals used for scientific purposes and animal experimentation.

## Conflicts of Interest

The authors declare no conflicts of interest.

## Data Availability

The data that support the findings of this study are available from the corresponding author upon reasonable request.
